# Advances and Trends in Real Time Visual Crowd Analysis

**DOI:** 10.3390/s20185073

**Published:** 2020-09-07

**Authors:** Khalil Khan, Waleed Albattah, Rehan Ullah Khan, Ali Mustafa Qamar, Durre Nayab

**Affiliations:** 1 Department of Electrical Engineering, University of Azad Jammu and Kashmir, Muzaffarabad 13100, Pakistan; 2 Department of Information Technology, College of Computer, Qassim University, Buraydah 51452, Saudi Arabia; w.albattah@qu.edu.sa (W.A.); re.khan@qu.edu.sa (R.U.K.); 3 Department of Computer Science, College of Computer, Qassim University, Buraydah 51452, Saudi Arabia; ali.mustafa@gmail.com; 4 School of Electrical Engineering and Computer Science, National University of Sciences and Technology, Islamabad 44000, Pakistan; 5 Department of Computer Systems Engineering, University of Engineering and Technology, Peshawar 25000, Pakistan; nayab.khan@uetpeshawar.edu.pk

**Keywords:** crowd image analysis, crowd monitoring, crowd management, deep learning, crowd detection

## Abstract

Real time crowd analysis represents an active area of research within the computer vision community in general and scene analysis in particular. Over the last 10 years, various methods for crowd management in real time scenario have received immense attention due to large scale applications in people counting, public events management, disaster management, safety monitoring an so on. Although many sophisticated algorithms have been developed to address the task; crowd management in real time conditions is still a challenging problem being completely solved, particularly in wild and unconstrained conditions. In the proposed paper, we present a detailed review of crowd analysis and management, focusing on state-of-the-art methods for both controlled and unconstrained conditions. The paper illustrates both the advantages and disadvantages of state-of-the-art methods. The methods presented comprise the seminal research works on crowd management, and monitoring and then culminating state-of-the-art methods of the newly introduced deep learning methods. Comparison of the previous methods is presented, with a detailed discussion of the direction for future research work. We believe this review article will contribute to various application domains and will also augment the knowledge of the crowd analysis within the research community.

## 1. Introduction

Crowd or mass gatherings at various venues such as entertainment events, airports, hospitals, sports stadiums, theme parks are faced by the individuals on almost a daily basis. The activities are quite diverse and range from social and cultural to religion. Unlike social and sports related events, the crowd situations experienced by the people on important religious events like Hajj and Umrah may not be possible to avoid. It is therefore important to have an intelligent Crowd Monitoring System (CMS) to ensure the safety of the public, maintain high throughput of pedestrians flow to prevent stampedes, provide better emergency services in case of crowd-related emergencies and to optimize the resources for providing good accessibility by avoiding congestion.

In general perspectives, crowd management, monitoring, and analytics have potential for a number of applications. These include but are not limited to the safety domain, emergency services, traffic flow and management in private and public spaces, people counting and analyzing group behaviors and similarly swarm-based applications. Such integrity of applications provides a natural demand for research and developments in managing and analyzing crowd and the behavior of individuals in crowd for groups analysis, counting and summarizing, density and prediction, flow analysis, specific behavior prediction, and mass tracking. In general, the group detection and density estimation have proven useful for corresponding steps of intelligent analytics and several applications. The review article [1] divides the counting of individuals into three categories; object-based counting, clustered counting, and regression-based counting.

Real time CMS has received increasing attention, especially during the last 10 years. Two reasons account for this in our proposed article. First, many image analysis tasks can benefit from efficient crowd management system, such as Hajj and Umrah [2,3,4,5,6,7,8] etc. Secondly, although some success of low level has been achieved in the last 10-15 years, crowd management is still challenging, particularly videos taken in unconstrained conditions. Due to the last two reasons, crowd management remains an open challenge, and various new state-of-the-art (SOA) methods have been proposed by researchers from time to time.

A crowd can increase in no time and controlling the crowd can become very challenging for the organizers. In such cases, problems such as abnormal behavior [9,10] or even stampedes could occur. The crowds can now be monitored in real time using Unmanned Aerial Vehicles (UAVs) and closed-circuit television (CCTV) cameras. While the CCTV cameras are in general limited, UAVs provide coverage of a much larger area, are fully mobile, and can provide high-resolution and real time images. Motlagh et al. [11] discussed a scenario of crowd surveillance using UAV and face recognition. Rather than local video data processing, the task was offloaded to a remote node. Similarly, Al-Sheary and Almagbile [12] have developed a crowd monitoring system using UAV. They used color segmentation to identify the pedestrians. Their method was able to identify the crowd accurately in case of Hajj. The color of feature classes is either black or white or one or two classes with mostly black and white. This made the identification task easier for the system.

Alotaibi et al. [13] developed a deep Convolutional Neural Network (CNN) based crowd counting technique using Internet of Things (IoT). The application was developed specifically for Saudi public places and was able to robustly count the people in low and highly crowded areas. A dataset containing 750 images with an average of 80 persons in each image was created. The images were collected from videos obtained from various places such as malls, restaurants, and airports.

Makkah is located in Saudi Arabia and is visited by millions of people every year. Whereas Umrah goes on throughout the year, Hajj is an annual ritual where Muslims visit Makkah and its surroundings, and is one of the largest gatherings of mankind. According to the General Authority for Statistics (GASTAT) in Saudi Arabia, more than 2.4 million performed Hajj in 2019 [14]. Circumabulating the Ka’aba, also known as Tawaf, is performed seven times and is a key component of both Hajj and Umrah. It is normally observed that the crowd density during Tawaf increases a lot during peak hours. Furthermore, kissing the Black Stone (a part of Tawaf) is a daunting task. Mohamed and Parvez [4] developed a model for real time crowd monitoring during Tawaf and presented a queue-based system to touch and kiss the Black Stone.

## 2. Applications

**Figure 1:**
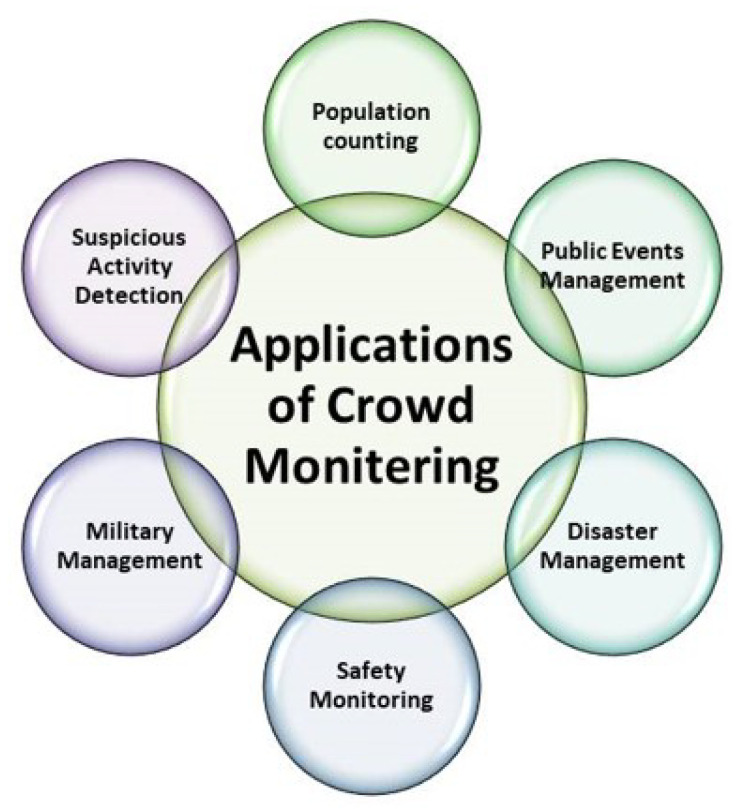
Applications of crowd monitoring and management system.

Several applications exhaustively rely on a robust and efficient crowd management and monitoring system. Applications of crowd monitoring and management system are summarized in Figure 1. In the following paragraphs, we discuss the tasks that highly depend on real time CMS:

- People counting in dense populated areas: Population of the world is growing day by day. Maintaining public order in certain crowded places such as airports, carnivals, sports events, and railway stations is very essential. In crowd management system, counting people is an essential factor. Particularly in smaller areas, increase in the number of people create problems such as fatalities, physical injury etc. Early detection of such kind of a crowd avoid these problems. In such sort of crowd management, counting the number of people provide accurate information about certain conditions such as blockage at some points and so on. Instead of large-scale research work, counting methods are still facing various challenges such as varying illumination conditions, occlusion problems, high cluttering, and some scale variations due to various perspectives. Due to a lot of development in the design of CMS, difficulties of people counting are now reduced to some extent. Some excellent works are proposed in [15,16,17,18,19], which address people counting through an efficient CMS.
- Public Events Management: Events such as concerts, political rallies, and sports events are managed and analysed to avoid specific disastrous situations. This is specifically beneficial in managing all available resources such as crowd movement optimization and spatial capacity [20,21,22]. Similarly crowd monitoring and management in religious events such as Hajj and Umrah is another issue to be addressed. Each year millions of people from different parts of the world visit the Mosque of Makkah for Hajj and Umrah. During Hajj and Umrah, Tawaf is an essential activity to be performed. In specific peak hours, crowd density in Mataf is extremely intense. Kissing the Black Stone in Hajj and Umrah is also a daunting task due to a large crowd. Controlling such a big crowd is a challenging task during Hajj and Umrah. An efficient real time crowd management system is extremely needed in such occasions. Some works which propose Hajj monitoring system can be explored in the papers [4,5,6,7,8,23,24,25].
- Military Applications: The number of fighting jets, soldiers, and moving drones and their motion etc. are estimated through proper crowd management systems. Thus the strength of the armed forces can be estimated through this system [26,27,28].
- Disaster Management: There are various overcrowding conditions such as musical concerts and sports events etc., where when a portion of crowd charges in random directions, causing life-threatening conditions. In past, large numbers of people died due to suffocation in crowded areas in various public gathering events. Better crowd management can be made in such events to avoid accidents [29,30,31].
- Suspicious-Activity Detection: Crowd monitoring systems are used to minimize terror attacks in public gatherings. Traditional machine learning methods do not perform well in these situations. Some methods which are used for proper monitoring of such sort of detection activities can be explored in [32,33,34,35].
- Safety Monitoring: A large number of CCTV monitoring systems are installed at various places such as religious gatherings, airports, and public locations which enable better crowd monitoring systems. For example, [36] developed a system which analyze behaviors and congestion time slots for ensuring safety and security. Similarly, [37] presents a new method to detect dangers through analysis of crowd density. A better surveillance system is proposed which generates a graphical report through crowd analysis and its flow in different directions [38,39,40,41,42,43].

## 3. Motivations

Efficient crowd monitoring and management contributes to various applications having further potential for computer vision (CV) paradigm; however, crowd management in real time is far from being solved, particularly in the wild conditions and still facing many open challenges. The literature also reports some success stories, and some convincing research work has also been reported, especially in the constrained conditions. However, under uncontrolled scenarios, the task of crowd management is still open for research community. Several factors contribute to a robust real time CMS and also affect the performance of an accurate CMS. Some of the factors include occlusions, changes in illumination conditions, noise in various forms, changes in facial expressions and head poses, etc. Moreover, the number of publicly available datasets for crowd management is minimal. There are only a few datasets available for research work. We summarize some of these challenges as follows:

- When two or more than two objects come close to each other and as a result merge, in such scenarios, it is hard to recognize each object individually. Consequently, monitoring and measuring accuracy of the system becomes difficult.
- A non-uniform sort of arrangement of various objects which are close to each other is faced by these systems. This arrangement is called clutter. Clutter is closely related to image noise which makes recognition and monitoring more challenging [43].
- Irregular object distribution is another serious problem faced by CMS. When density distribution in a video or image is varying, the condition is called irregular image distribution. Crowd monitoring in irregular object distribution is challenging [44].
- Another main problem faced in real time crowd monitoring systems is aspect ratio. In real time scenarios, normally a camera is attached to a drone which captures videos and images of the crowd under observation. In order to address the aspect ratio problem, the drone is flown at some specific height from the ground surface and installation of the camera is done such that the camera captures the top view of the crowd under observation. The top view results in properly addressing the aforementioned problem of aspect ratio.

In machine learning tasks that are based on specific model learning paradigms, the availability of data for training and testing is of crucial importance and an essential requirement of the success of a particular task. The unavailability of a public dataset is one major problem towards the development of an efficient and mature real time CMS. Although datasets are available for counting purposes, but very few datasets are available for behavior analysis and localization research. In addition, over the last 10 years, some excellent methods have been introduced and developed for CMS; however; research community still need immense efforts to contribute and develop an optimal and accurate real time CMS. Such issues, factors, and variables in SOA motivate us to address the crowd management area with interest and analyse the approaches, developments, applications, and future directions in the crowd management domain. Moreover, the shift from traditional to deep learning approaches motivates us for a comprehensive and an up-to-date review, which will help researchers and also contribute to numerous applications and domains.

## 4. Contributions

In this paper, we present a detailed review of crowd management systems, focusing on methods for both controlled and uncontrolled environmental conditions. We present merits and demerits of SOA approaches by focusing on seminal work, and then culminating SOA methods that are based on deep learning frameworks. A comparison of the previous methods leads us to the potential future directions for research on the topic. We believe that such a single review article will recap and contribute to various application domains, and will also augment the topic knowledge of the research community.

Our proposed article is combining literature on the topic over the last 10 years. We focus particularly on SOA CMSs that have been introduced over the last 10 years. We also focus on the shift occurring in SOA towards the new paradigm of the deep learning methods from traditional machine learning methods.

We organize the rest of the paper as follows: Section 5 provides a description of different databases available for CMS. Section 6 presents the crowd management and monitoring methods reported so far. Section 7 gives a detailed comparison of SOA methods reported to date. Finally, we conclude the paper in Section 8 with a fruitful discussion and potential future directions.

## 5. Databases

The performance of the CMS is evaluated with available crowd datasets. Crowd management is a relatively less explored area with less publicly available data. Most of the datasets have one or sometimes two scenes, hence cannot be used for generic crowd understanding. In this section, we discuss the available crowd monitoring databases for the topic. The datasets are available in the form of videos and images. A summary of the datasets is presented in Table 1.

- Mecca [45]: This dataset is collected in the holy city of Makkah, Saudi Arabia during Hajj. Million of Muslims pray around the central Kaaba during Hajj each year. The duration of the video clip is short and for 10 min only. The video clip records specific period when pilgrims enter the Kaaba and occupy the place. The cameras are fixed at three specific directions, including south, east, and west. Video synchronization is performed for all the three cameras, as the videos are recorded from multiple cameras. The starting date and time is recorded along with other information. The ground truth data are created through labelling of pedestrians in every grid. The total number of frames in Mecca dataset is 480, whereas 8640 grid images are obtained.
- Kumbh Mela [46]: The Kumbh Mela dataset is collected with a drone camera for the holy Hindu festival at Allahabad, India. The dataset can be used both for crowd counting and modelling at religious gatherings. Kumbh Mela is a mass Hindu pilgrimage which is held after each 12 years. The Hindus gather at the confluence which is also called Sangam, invisible Saraswati, and Yamuna. In the last festival, held in 2013 a crowd of 120 million people was observed. All videos in Kumbh Mela are collected in a densely populated area. A drone with camera flying above the crowd captures video frames in this dataset. This is a large dataset which is recorded for around 6 h consisting of 600K frames.
- NWPU-crowd [47]: Most of the publicly available datasets are small-scale and cannot meet the needs of deep convolutional neural networks (DCNNs) based methods. To solve this problem, a large-scale dataset which can be used both for crowd counting and localization has been recently introduced. The NWPU-Crowd dataset consists of around 5K images where 2,133,375 heads are annotated. Compared to other datasets, NWPU-Crowd has large density range and different illumination scenes are considered. The data are collected both from Internet and self shooting. A very diverse data collection strategy has been adapted; for example, some of the typical crowd scenes are: resorts, malls, walking street, station, plaza, and museum.
- Beijing Bus Rapid Transit (BRT) [48]: The Beijing BRT database contains 1280 images captured from surveillance cameras fixed at various Beijing bus stations. The authors of the paper fixed the data for the training and testing phases; 720 images are used for training and remaining for testing phases. To make the database complex; shadows, sunshine interference, glare, and some other factors were also included.
- UCF-QNRF [49]: The latest dataset introduced is UCF-QNRF which has 1535 images. The dataset has massive variation in density. The resolution of the images is large (400 × 300 to 9000 × 6000) as compared to SOA datasets. It is the largest dataset used for dense crowd counting, localization, and density map estimation, particularly for the newly introduced deep learning methods. The images for UCF-QNRF are collected from the Hajj footage, flickr, and web search. The UCF-QNRF has the highest number of crowd images and annotation is also provided. A large variety of scenes containing very diverse set of densities, viewpoints and lighting variations are included. The UCF-QNRF contains buildings, sky, roads, and vegetation as present in realistic scenes captured in the unconstrained conditions. Due to all these conditions, the dataset is more difficult and realistic as well.
- The Shanghai Tech. [50]: This dataset has been introduced for comparatively large scale crowd counting. The dataset contains 1198 images whereas annotated heads are 330,165. This is one of the largest datasets as the number of annotated heads are sufficiently large for evaluation and training. The Shanghai dataset consists of two parts, namely Part A and B. Part A consists of 482 images, which are taken from the Internet; whereas Part B consists of 716 images which are collected from the metropolitan street in the city of Shanghai. The training and evaluation sets are defined by the authors. Part A has 300 images for training and remaining 182 for evaluation. Similarly, Part B has 400 images for training and 316 for the testing phases. The authors of the dataset attempt to make the dataset challenging as much diversity is included with diverse scenes and varying density levels. The training and testing phases are very biased in the Shanghai dataset as the images are of various density levels and are not uniform.
- WorldExpo [44]: This dataset is used for cross scene crowd management scenarios. The dataset consists of 3980 frames having size 576×720. The total number of labeled pedestrians is 199,923. The authors in WorldExpo dataset perform data drive cross scene counting in a crowded scene. All videos are collected through 108 cameras which are installed for surveillance applications. Diversity is ensured in the scenes as videos are collected from cameras with disjoint bird views. The training set consists of 1127 1-min long videos from 103 scenes and testing set consists of five 1-h videos which are collected from five different scenes. Due to limited data, the dataset is not sufficient for evaluating approaches designed for dense crowded scenes.

WWW [51]: The dataset Who Do What at Some Where (WWW) is particularly designed for densely crowded scenes. This dataset is collected from very diverse locations such as shopping malls, parks, streets, and airports. The WWW consists of 10,000 videos captured from 8257 different scenes with eight million frames. The dataset contains data from almost all real world scenarios. The authors of the paper further define 94 attributes for better elaboration of the data. Specific keywords are used to search for videos from different search engines including YouTube, Pond, and Getty Images.

- UCF_CC_50 [43]: It is comparatively a difficult dataset as various scenes and different varieties of densities are considered. The database is collected from various places such as stadiums, concerts, and political protests. The total number of annotated images is 50 whereas the number of individuals is 1279. Only limited images are available for evaluation and training phases. The individuals varies from 94 to 4543, showing large-scale variations across the images. As the number of images for training and testing are limited, cross validation protocol is adapted for training and testing phases by the authors. Both 10-fold and 5-fold cross validation experiments are performed. Due to its complex nature, the results reported so far on recent deep learning based methods on this database are still far from optimal.
- Mall [52]: The Mall dataset is collected through surveillance cameras which are installed in a shopping mall. The total number of frames is the same as in University of California at San Diego (UCSD, whereas the size of each frame is 320×240. As compared to UCSD, little variation in the scenes can be seen. The dataset has various density levels and different activity patterns can also be noticed. Both static and moving crowd patterns are adapted. Severe perspective distortions are present in the videos, resulting in variations both in appearance and sizes of the objects. Some occlusion is also present in the scene objects such as indoor plants, stall etc. The training and testing sets are defined in the Mall dataset as well. The training phase consists of first 800 frames whereas remaining 1200 frames are used for testing.
- PETS [53]: It is comparatively an old dataset, but is still used for research due to its diverse and challenging nature. These videos are collected through eight cameras which are installed in a campus. The dataset is used for surveillance applications, consequently complex videos can be seen. The dataset is mostly used for counting applications. Labelling is provided for all video sequences. PETS contains three kinds of movements and further each movement includes 221 frame images. The pedestrian level covers light and medium movements.
- UCSD [54]: The UCSD dataset is the first dataset which is used for counting people in a crowded place. The data in UCSD are collected through a camera which is installed on a pathway specified for pedestrians. All the recording is done at the University of California at San Diego (UCSD), USA. Annotation is provided for every fifth frame. Linear interpolation is used to annotate the remaining frames. To ignore unnecessary objects (for example trees and cars etc.), a region of interest is also defined. The total number of frames in the dataset is 2000, whereas the number of pedestrians is 49,885. The training and testing sets are defined, the training set starting from indices 600 to 1399, whereas testing set contains remaining 1200 sequences. The dataset is comparatively simple and an average of 15 people can be seen in a video. The dataset is collected from a single location, hence less complexity can be seen in the videos. No variation in the scene perspective across the videos can be noticed.

**Figure 2:**
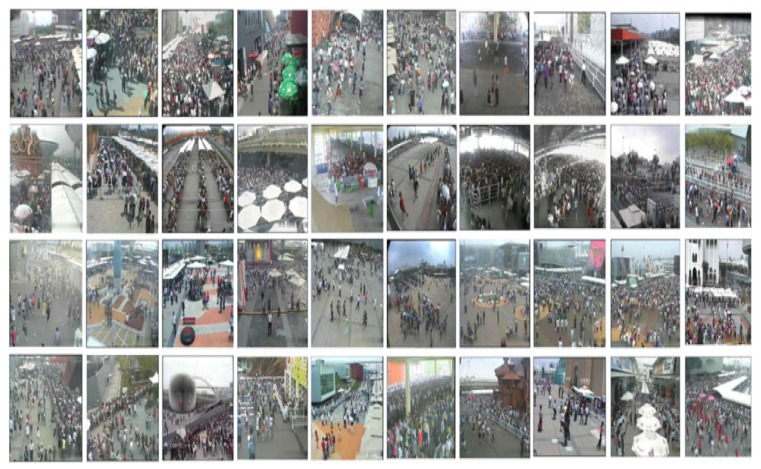
Some sample images from World Expo dataset.

**Figure 3:**
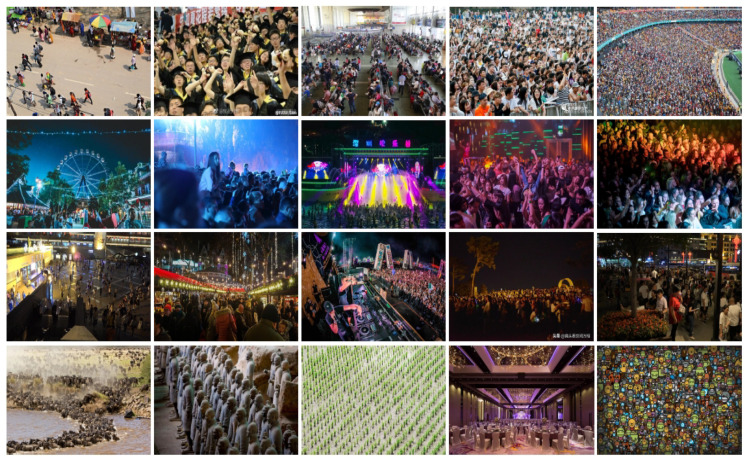
Some sample images from NWPU-counting dataset.

**Figure 4:**
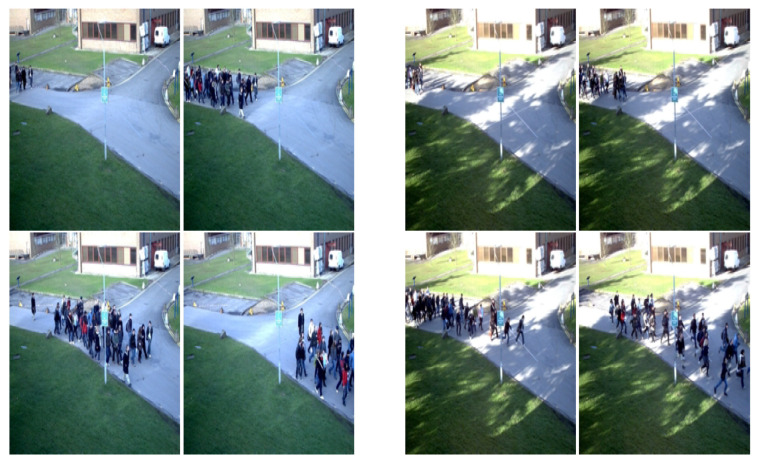
Some sample images from PETS dataset.

Some sample images from these dataset are shown in Figure 2, Figure 3 and Figure 4. The datasets are summarized in Table 1.

## 6. Approaches

Counting of crowd provides an estimate about the number of people or certain objects. Counting does not provide any information about the location. Density maps are computed at different levels and also provide very weak information about a person’s location. On the other hand, localization provides accurate information about the location. However, due to sparse nature, it is comparatively a difficult task. Therefore, the best way is to handle all the three tasks simultaneously, employing the fact that each case is related to the other.

**Figure 5:**
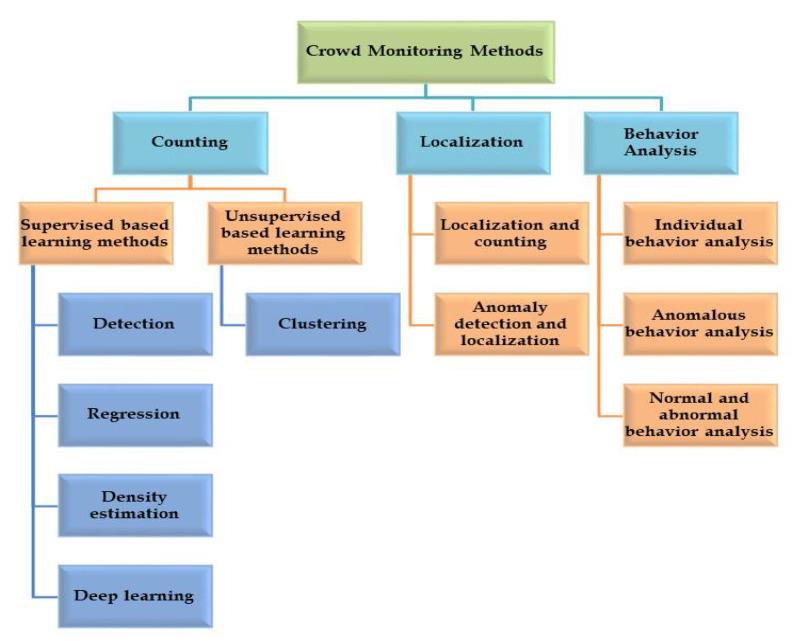
Crowd monitoring methods reported in literature.

We discuss various methods that are used to address crowd controlling and management system in this section. We do not claim any generic taxonomy for CMS; instead, we organize each real time CMS based on the fundamental method that underlines its implementation. We also discuss sufficient references where these proposed methods are previously used. We present discussion regarding the merits and demerits of each method as well. A summary of all the methods reported by literature is presented in Figure 5.

We make three categories of crowd monitoring including; localization, behaviour, and counting. Then each of these categories are further divided.

### 6.1. Localization

We divide localization into two sub categories including localization and counting and anomaly detection. Rodriguez et al. [55] propose a method for localizing crowded scenes using density maps. The authors of the paper optimize the objective function which prefers those density maps which are generated on specific detected locations, almost similar to the estimated density map [56]. Better precision and recall values are obtained with this approach. A Gaussian kernel is placed at the location of detection and the density map is generated. A density map is obtained by Zheng et al. [57] through sliding window over the image [56]. In the later stage, integer programming is used for localizing objects on density maps. Similarly, Idrees et al. [43] present a method for crowd analysis, addressing all the three terms including counting, density estimation, and localization through composition loss function. The formulation in [43] work is based on an observation that all the three tasks are related to each other which makes the loss function for better optimization of a DCNNs decomposable. As localization needs comparatively better quality images, a new dataset known as UCF-QNRF is also introduced by the authors. Some papers recently introduced addressing anomaly detection can be addressed in the references [58,59,60].

### 6.2. Crowd Behaviour Detection

Behaviour analysis of large crowd has become the primary part for peaceful events organization [61]. In video processing particularly, behaviour analysis and identification is of crucial importance [10]. The researchers proposed various algorithms from time to time. The authors in [10,62] use optical flow to detect the behaviour of crowd. Another method in [63] use optical flow along with support vector machine (SVM) for crowd behaviour analysis. Similarly, [64] uses a deep learning method with optical flow for crowd behaviour detection. Some additional methods which use Isometric Mapping [65], spatio-temporal [66] and spatio-temporal texture [44] can also be explored for details.

### 6.3. Counting

Gathering of people for some specific reason such as political gathering, religious occasion, and sports event is called crowd. Estimating the number of people in videos or images is called crowd counting. We divide crowd counting into two types, known as supervised and unsupervised counting. In the first type of counting, the input data are normally labeled and then some machine learning tool is used for prediction. In unsupervised crowd counting, the data and labels are unknown. A machine learning tool is used for categorization. These two categories are further divided into other types as shown in Figure 5. The supervised crowd counting is further divided into the following types:

- Supervised learning based methods:
  – Counting by detection methods: A window of suitable size slides over the entire scene (video/image) to detect people. After detection, researchers came up with various methods using the concepts of histogram of oriented gradients (HOG) [67], shapelet [68], Haar features [69], and edgelet [70]. Various machine learning strategies are exploited by researchers [71,72], but most of these methods fail over highly crowded scenes. An excellent 3D shape modeling is used by Zhao et al. [73], reporting much better results as compared to SOA. The same work is further enhanced by Ge and Collins [74]. Some papers addressing counting by detection methods can be explored in the references [75,76,77].
    These methods fail when the density of crowd is high. Similarly, the performance of detection-based methods drop when a scene is highly cluttered.
  – Regression based method: The high density and cluttered problem faced by the aforementioned method is excellently addressed by this method. Regression based methods work in two steps: feature extraction and regression modelling. The feature extraction methods include subtraction of background, which is used for extracting the foreground information. Better results are also reported while using Blobs as a feature [39,54,78]. Local feature include extracting edge and texture information from data. Some of the local features used are Gray level co-occurrence matrices (GLCMs), Local binary pattern (LBP), and HoG. In the next stage mapping is performed from the extracted features through regression methods including Gaussian process regression, linear regression, and ridge regression [79]. An excellent strategy is adapted by Idrees et al. [43] by combining Fourier transform and SIFT features. Similarly, Chen et al. [39] extract features from sparse image samples and then mapping it to a cumulative attribute space. This strategy helps in handling the imbalanced data. Some more methods addressing counting problem can be explored in [15,16,17,39,80].
    The occlusion and cluttering problems faced by the initial two methods are solved with regression based methods. However, these methods still face the capitalized spatial information issue.
  – Estimation: A method incorporating the spatial information through linear mapping of local features is introduced by Lempitsky et al. [56]. The local patch features are mapped with object density maps in these methods. The authors develop the density maps by a convex quadratic optimization through cutting plane optimization algorithm. Similarly, Pham et al. [40] suggest a non-linear mapping method through Random Forest (RF) regression from patches in the image. The lastly mentioned method solve the challenge of variation invariance faced previously. Wang and Zou’s [38] work explores the computational complexity problem through subspace learning method. Similarly, Xu and Qiu [81] apply RF regression model for head counts. Some more algorithms which are estimation based methods can be explored in [56,82].
    We divide the density-level algorithms into three more categories:
    Low-level density estimation methods: These algorithms include methods such as optical flow, background segmentation method, and tracking methods [83,84]. These methods are based on motion elements. These elements are obtained from frame by frame modeling strategy, which is paving the path for object detection. Some more low density methods can be explored in [85,86,87].
    Middle-level density estimation methods: At this mid level of density estimation, the patterns in data become dependent upon the classification algorithms.
    High-level density estimation methods: In high level density estimation techniques, dynamic texture models are utilized [88]. These methods are dominant crowd modeling methods.
- Deep learning based methods (DLMs): As compared to TMLMs, recently introduced DLMs brought a large improvement in performance in various visual recognition tasks [89,90,91,92,93]. The TMLMs are based on handcrafted features, whereas, DLMs are more engineered. Apart from TMLMs, DLMs are also explored by researchers to address the counting problem in crowd. Wang et al. [47] perform experiments over AlexNet in dense crowded scene. Similarly Fu et al. [94] classify images into five levels considering the density in each image. The five levels defined by the authors include high density, very high density, low density, very low density, and medium density. Similarly Walach and Wolf [95] present a cross counting model. The residual error is estimated in the proposed model by adding layered boosting CNNs into the model. The method also performs selective sampling which reduces the effect of low quality images such as outliers. Zhang et al. [50] suggests a DCNNs multi-column based method for crowd counting. To cater various head sizes, three columns with various filter sizes are used. Similarly, Li et al. [96] use dialated DCNNs for better understanding of deeply congested scenes. Zhang et al. [73] present another crowd counting method through scale-adaptive DCNNs. To provide a regression based model, the authors suggest a multi-column DCNN model. Another method proposed in [97] use spatio-temporal DCNNs for counting in a crowded scene in videos. Another regression based model is proposed by Shang et al. [98]. Similarly Xu et al. [81] utilize the information at much deeper level for counting in complex scenes.

- Unsupervised learning based methods:
  – Clustering: These methods rely on the assumption that some visual features and motion fields are uniform. In these methods, similar features are grouped into various categories. For example, the work proposed in [18] uses Kanade–Lucas–Tomasi (KLT) tracker to obtain the features. The extracted features are comparatively low level. After extracting the features, Bayesian clustering [99] is employed to approximate the number of people in a scene. Such kind of algorithms model appearance-based features. In these methods, false estimation is obtained when people are in a static position. In a nutshell, clustering methods perform well in continuous image frames. Some additional methods are in the references [18,99,100,101].

Crowd counting and abnormal behavior detection are among the hottest issues in the field of crowd video surveillance. In the SOA, several articles discuss abnormal behavior detection in the crowd. To the best of our knowledge, it can be divided into two main categories, which are the global representation and local exceptions. The authors in [102] report two novelties for abnormal behavior detection. First, the texture extraction algorithm based on the spatial-temporal is developed. The second novelty is the approach for motion patterns of the crowd for identifying the unusual events in the crowd. These are termed as the signatures. An enhanced gray level co-occurrence matrix is employed for these signatures. The authors report superior performance compared to other approaches. For a crowd, abnormal events detection, the research in [103] considers both the appearance and motion flow information. Swarm theory-based Histograms of Oriented Swarms (HOSs) is introduced as a novelty. The HOS creates a signature for the crowded environments dynamics. The features of motion and appearance are employed only for local noise suppression, performance increase for non-dominant detection of local anomalies, and lowering the processing cost. As such, the approach gets an increased accuracy for pixel-based event recognition in the crowd. Ref. [104] proposes a Point-based Trajectory Histogram of Optical Flow (PT-HOF) for abnormal event detection in crowded environments. The (PT-HOF) captures the temporal and spatial info for the point trajectory in the scenes of crowd. It encodes the relevant features using the deep learned model. The work in [15] proposes the Markov Random Field (MRF), taking into account the space-time peculiarities. The local regions in video sequences are represented by the nodes in the graph of the MRF. The links in the MRF graph correspond to the neighbouring nodes in space-time. For normal and abnormal activities, the authors employ the optical flow, taking advantage of the probabilistic PCA. The model thus optimally captures the normal and abnormal actions locally and globally. The authors present an integrative pipeline approach in [16]. The approach integrates the output of the pixel analysis and the trajectory analysis for the normal and abnormal events differentiation. The normal and abnormal behaviours are detected based on the trajectories and speeds of objects, taking into account the complex actions in sequences. The work in [17] presents three attributes for localized video-based approaches for anomaly detection in sequences. Firstly, augmenting the dynamics and appearance of the scene and its detection ability. Second and third, are temporal- and spatial-based abnormal events. The approach is demonstrated to outperform existing methods. In [18], local motion-based video descriptors are used for feature extraction for abnormal events modeling, achieving superior accuracy in localization tasks, and video abnormal events detection. The work in [19] uses the motion history for consecutive frames in sequences for anomalies detection. These motion histories are termed as the Short Local Trajectories (SLTs). The SLTs are extracted from the super-pixels of the foreground objects in the scene. The SLT thus encodes the temporal and spatial information of the moving subjects. The authors report the feasibility of the approach on three datasets. Concerning the global anomalies, the authors in [4] present a framework that takes into account the Spatio-temporal structure of the sequences. The framework thus exhibits an optimal decision rule. For the local anomalies, the local optimal decision rules are extracted. This optimal local decision rules even work when the behavior has spatial, global, and temporal statistical properties and dependence. For abnormal and normal events differentiation, the authors in [5] present the Sparse Reconstruction Cost (SRC). By using each basis before weight, the SRC provides a robust generalization of the vents in normal and abnormal classes. In [7], a novel approach in three aspects is demonstrated. For modelling of crowded scenes, the approach uses the particle trajectories. Secondly, for crowd motion capturing and modelling, the authors introduce chaotic dynamics. Finally, for abnormal events detection, a probabilistic model is formulated. The results show that the proposed approach efficiently model, recognize, and differentiate normal and abnormal events in sequences.

Crowd video surveillance is not limited to crowd counting and anomaly detection, and many new directions have been expanded, such as salient detection, congestion detection, etc. Saliency detection refers to the process of imitating the human visual system while using computer vision methods. Nguyen et al. [105] use the knowledge-driven gaze in human visual system to find the saliency in crowd. They used CNN using self-attention mechanism so as to find the salient areas in human crowd images. Similarly, Zhang et al. [106] were able to detect salient crowd motion using direction entropy and a repulsive force network. The frames of the crowd video sequence are evaluated by an optimal flow technique. This is followed by the calculation of the crowd velocity vector field. The authors worked on three video sequences from the Crowd Saliency dataset such as a train station scene, marathon scene, and Hajj pilgrimage scene. Retrograde and instability areas of a crowd were identified. In the paper by Lim et al. [107], the authors discuss how the temporal variations in the flow of a crowd could be exploited to identify the salient regions. The salient regions have high motion dynamics and are found in different scenarios such as occlusions, evacuation planning at entry and exit points, identification of bottlenecks. In an irregular flow, the motion dynamics of people differ from one another. For Mecca, their method identified the salient regions produced by the bottlenecks which were observed near Black Stone and the Yemeni corner. Furthermore, their method does not need tracking each object separately or prior learning of the scene. Lim et al. [108] were able to identify the salient regions in crowd scenes using an unsupervised algorithm. Their approach identified the crowding sources and sinks corresponding to areas in a scene where the people in a crowd enter and exit respectively. They detect the salient motion regions through ranking the intrinsic manifold obtained by similarity feature maps. Khan studied the individuals struck in congested areas of a crowd [109]. Such individuals experience lateral oscillations and are unable to move in a free manner. The pedestrians trajectories are used to determine the oscillation feature. An oscillation map is used to find the critical locations and congestion in videos. Furthermore, a novel dataset consisting of 15 crowd scenes to evaluate congestion detection methods was proposed.

## 7. Results and Discussion

### 7.1. Quantification of Tasks

- Counting:
  We represent estimation of count for crowded image *i* by $c_{i}$. This single metric does not provide any information about the distribution or location of people in a video or image, but is still useful for various applications such as predicting the size of a crowd which is spanning many kilometres. A method proposed in [110] divides the whole area into smaller sections, which further finds the average number of people in each section, and also computes the mean density of the whole region. However, it is extremely difficult to obtain counts for many images at several locations, thereby, the more precise integration of density over specific area covered is permitted. Moreover, cartographic tools are required for counting through aerial images which map the crowd images onto the earth for computing ground areas. Due to its complex nature, mean absolute error (MAE) and mean squared error (MSE) are used for evaluation of a crowded scene for counting.
  The two evaluation metrics MAE and MSE can be defined as;
  (1) $$MAE=\frac{1}{N}\sum_{i=1}^{N}\left|X_{i}-X_{i}^{\prime }\right|$$
  (2) $$MSE=\sqrt{\frac{1}{N}\sum_{i=1}^{N}{\left|X_{i}-X_{i}^{\prime }\right|}^{2}}$$
  In Equations (1) and (2), N represents the number of test samples, $x_{i}$ the ground truth count, and $x_{i}^{\prime }$ the estimated count for the *i*th sample.
- Localization: In many applications, the precise location of people is required, for example, initializing a tracking method in high density crowded scene. However, to calculate the localization error, predicted location is associated with ground truth location by performing 1-1 matching. This is performed with greedy association and then followed by computation of Precision, Recall, and F-measure. Moreover, the overall performance can also be computed through area under the Precision-Recall curve, also known as L-AUC.
  We argue here, precise crowd localization is comparatively less explored area. Evaluation metrics of localization problem are not firmly established by researchers. The only work which proposes 1-1 matching is reported in [43]. However, we observe that the metric defined in [43] leads to optimistic issues in some cases. No penalizing has been defined in over detection cases. For instance, if true head is matched with multiple heads, the nearest case will only be kept while ignoring the remaining heads without receiving any penalty. We believe that for a fair comparison, the discussed metric fails to be acknowledged widely. We define all the three evaluation metrics as:
  (3) $$Precesion=\frac{t_{p}}{t_{p}+f_{p}}$$
  (4) $$Recall=\frac{t_{p}}{t_{p}+f_{n}}$$
  (5) $$F-measure=2*\frac{Precision\times Recall}{Precision+Recall}$$
  where $t_{p}$ represents true positive and $t_{n}$ represents false negative. For crowd localization task, normally box level Precision, Recall, and F-measure is used.
- Density estimation: Density estimation refers to calculating per-pixel density at a particular location in an image. Density estimation is different from counting as an image may have counts within particular safe limits, whereas containing some regions which will have comparatively higher density. This may happen to some empty regions located in a scene such as sky, walls, roads etc. in aerial cameras. The metrics which were used for counting estimation were also used for density estimation, however, MAE and MSE were measured on per pixel basis.

### 7.2. Data Annotation

Tools: Annotation is a process of creating ground truth data for a machine learning task. The data may be in the form of images, video, audio, text etc. The ground truth data are used by a computer to recognize patterns similar in an unseen data. The annotation categories are different such as line annotation, 3D cuboids, bounding box annotation, landmark annotation, and dot annotation. In crowd counting scenarios, dot annotation was the initial step which created ground truth and was carried through different tools such as LabelMe, RectLabel, LabelBox etc.

An online annotation tool was developed based on Java, HTML, and Python. This tool creates ground truth data for labelling head points. The tool normally supported two kinds of labels, bounding box and point. Each image was zoomed to label head with desired scales and was then divided into small patches of size 16×16. This size allowed annotators to create ground truth under five different scales $\left(2^{i},i=0,1,2,3,4\right)$ times original image size. This tool prompted the annotation process with good speed and much better quality. For more information, we would request the readers to explore the paper in [43].

Point wise annotation: The annotation process could be divided into two sub-stages, labelling and then performing refinement. Normally, some annotators were involved in the labelling process. This method of creating the ground truth data was a time consuming task, since a single person was involved in all labelling. After creating ground truth, some other individuals did the the preliminary annotation which took comparatively lesser time.

Annotation at box-level: The box-level annotation was performed in three steps. First, for each image, normally 10–20% points were typically selected to draw a bounding box. Secondly, for those points which were without a box label, a linear regression method was adapted to obtain its nearest box and box size as well. In the last stage, manual refining of the predicted box labels was performed.

In a nutshell, creating ground truth labels were mostly produced through a manual process. This labelling was performed without automatic labelling tool. Such a kind of labelling was totally dependent on the subjective perception of a single individual who was involved in this labelling task. Hence providing an accurate ground truth label in the image was very difficult and a time consuming task.

### 7.3. Comparative Analysis

We performed comparison of the existing SOA approaches on crowd management datasets. All results are summarized in Table 2 and Table 3. We summarize some concluding remarks in the following paragraphs.

- In the last few years, significant research work has been reported in the area of crowd analysis. This can be seen from Table 2, Table 3 and Table 4. Many datasets have been introduced. However, most of these datasets address the counting problem. Less focus has been given to localization and behaviour analysis. The only datasets having sufficient information about localization and behaviour analysis are UCF-QNRF and NWPU-crowd. Therefore, there is still a lot of space regarding publicly available datasets in crowd analysis.
- Most of the labelling for creating ground truth data was performed manually. Commercial image editing softwares were used by the authors for creating ground truth data. In such kind of labelling process, no automatic tool was used. This labelling was totally dependent on subjective perception of a single participant involved in labelling. Hence, chances of error exist. Differentiation of certain regions in some cases was difficult.
- As compared to counting and behaviour analysis, localization is a less explored area. Some authors report 1-1 matching [43]. However, we believe that the metric defined in [43] leads to some optimistic problems. In this metric, no penalizing strategy has been defined in cases where multiple head detection occurs. Hence, still a proper performance metric has not been defined for behaviour analysis.
- Crowd analysis is an active area of research in CV. Table 4 shows a summary of the research conducted on crowd analysis between 2010 to 2020. A more detailed picture is presented in Table 2 and Table 3, as more detailed results are shown. The MAE, MSE, Precision, Recall, and F-1 measure values are reported from the original papers. As can be seen from Table 2 and Table 3, all the metric values were improved on the standard database, particularly with recently introduced deep learning method.
- Some papers report that a more detailed look into the crowd counting, localization, and behaviour analysis reveal that traditional machine learning methods perform better in some cases as compared to newly introduced deep learning based methods. Through this comparison, we do not claim that the performance of hand-crafted features is better than deep learning. We believe that better understanding of the deep learning based architectures is still needed for crowd analysis task. For example, most of the cases of poor performance while employing deep learning were limited data scenarios, a major drawback faced by deep learning based methods.
- Deep learning based algorithms have shown comparatively better performance in various visual recognition applications. These methods have particularly shown improvement in more complex scenarios in image processing and CV. Most of the limitations of the traditional machine learning methods are mitigated with these methods. Just like other applications, crowd monitoring and management has also shown significant improvements in the last 10 years.
- The performance of conventional machine learning methods was acceptable with data collected in simple and controlled environment scenes. However, when these methods are exposed to complex scenario, significant drop in performance was observed. Unlike these traditional methods, deep learning based methods learn comparatively higher level of abstraction from data. As a result, these deep learning based methods outperform previous methods by a large margin. These methods reduce the need of feature engineering significantly. However, these deep learning based methods are also facing some serious concerns from the research community. For example, deep learning is a complicated procedure, requiring various choices and inputs from the practitioner side. Researchers mostly rely on a trial and error strategy. Hence, these methods take more time to build as compared to the conventional machine learning models. In a nutshell, deep learning is the definitive choice for addressing the crowd management and monitoring task properly, but till date the use of these methods is still sporadic. Similarly, training a deep learning based model for crowd monitoring with different hidden layers and some filters which are flexible is a much better way to learn high level features. However, if training data are not sufficient, the whole process may under perform.
- We notice that DCNNs model with relatively more complex structure cannot deal with multi-scale problem in a better way, and still improvement is needed. Moreover, the existing methods have more focus on the system accuracy, whereas the correctness of density distribution is ignored. From the results, we notice that the reported accuracies are more close to the optimal ones, as the number of false negative and false positive are nearly the same.
- We argue that most of the existing methods for crowd monitoring and management are using CNNs based methods. However, these methods employ the pooling layer, resulting in low resolution and feature loss as well. The deeper layers extract the high level information, whereas the shallower layers extract the low level features including spatial information. We argue that combining both information from shallow and deep layers is the better option to be adapted. This will reduce the count error and will generate more reasonable and acceptable density map.
- Traditional machine learning methods have acceptable performance in controlled laboratory conditions. However, when these methods were applied to datasets with unconstrained and un-controlled conditions, significant drop in performance is noticed. However, deep learning based methods show much better performance in the wild conditions.
- Crowd analysis is an active area of research in CV. Tremendous progress has been seen in the last 10 years. From the results reported till date, it is clear that all the metrics (MAE, MSE, F-1 measure) are improved. We present a summary of all the papers published in Table 2 and Table 3. Noting the fast trends of the CV developments moving very rapidly towards recently introduced deep learning, progress in crowd analysis is not satisfactory. Given the difficulty of the training phase in deep learning based methods, particularly crowd analysis, knowledge transfer [111,112] is an option to be explored in future. In knowledge transferring strategy, benefits from the models already trained are taken. We also add here that a less investigated domain in transfer knowledge is heterogeneous strategy adoption considering deep learning based techniques for crowd analysis, the keywords are temporal pooling, 3D convolution, LSTMs, and optical flow frames. Similarly, better managed engineering techniques are also needed to improve SOA results. For instance, data augmentation is another possible option to be explored.

## 8. Summary and Concluding Remarks

Crowd image analysis is an essential task for several applications. Crowd analysis provides sufficient information about several tasks including counting, localization, behaviour analysis etc. Crowd analysis is extremely challenging when data are collected in the wild conditions. However, some good research work particularly in the last 5 years reveals many achievements. Due to a diverse range of applications, we believe that crowd analysis in the present stage is far beyond the grasp, therefore, we call all researchers to improve the existing methods presented in Section 6.

One major problem crowd analysis is facing is the unavailability of a database for some tasks such as crowd localization and behaviour analysis. We expect from the research community of CV some contribution in the form of challenging datasets on the topic. We are also expecting excellent evaluations of the deep learning techniques, particularly, data collected in the un-constrained conditions in the form of future work. If an efficient crowd analysis system is introduced, the system will have profound effects on very large scale applications of crowd image monitoring systems.

We present a detailed survey on the crowd analysis methods, including details about all available databases. We also investigate various aspects of the already existing solutions for crowd analysis. We started from a hand crafted representation and moved towards newly introduced deep learning based techniques. Lastly, we provide comparative analysis of the obtained results so far for crowd image analysis. We also identify some open problems in crowd analysis and present an outlook into the future of crowd image analysis. 

## Figures and Tables

**Table 1:** Major Crowd Monitoring System (CMS) datasets.

Database	Year	Task	# of Images	Head Count	Source Obtained
Mecca [45]	2020	crowd monitoring	–	–	surveillance
Kumbh Mela [46]	2020	crowd monitoring	6k	–	surveillance
NWPU-Crowd [47]	2020	crowd counting and localization	5109	2133,375	surveillance and Internet
BRT [48]	2018	crowd monitoring	1280	16795	surveillance
UCF-QNRF [49]	2018	counting in a crowd and localization	1525	1,251,642	surveillance
Shanghai Tech [50]	2016	cross scene crowd counting	482	241,677	surveillance and Internet
WorldExpo’10 [44]	2015	counting in a crowd	3980	199,923	surveillance
WWW [51]	2015	crowd management	10000	8 million	Internet
UCF_CC_50 [43]	2013	Density estimation	50	63,974	surveillance
The Mall [52]	2012	counting in a crowd	2000	62,325	surveillance
PETS [53]	2009	counting in a crowd	8	4000	surveillance
UCSD [54]	2008	counting in a crowd	2000	49,885	Internet

**Table 2:** CMS performance in the form of Average Precision, Recall, F1-measure, and AUC metrics.

Database	Year	Method	Precision	Recall	L-AUC	F1-Measure
	2015	Ren et al. [113]	95.8	3.5	–	6.7
NWPU-crowd	2017	Hu et al. [114]	52.9	61.1	–	56.7
	2019	Gao et al. [115]	55.8	49.6	–	52.5
	2019	Liu et al. [116]	66.6	54.3	–	59.8
	2018	Idrees et al. [43]	75.8	63.5	0.591	–
	2015	Badrinarayanan et al. [117]	71.8	62.9	0.67	
	2016	Huang et al. [118]	70.1	58.1	0.637	
	2016	He et al. [119]	61.6	66.9	0.612	
UCF_QNRF	2018	Shen et al. [120]	75.6	59.7	–	–
	2018	Liu et al. [116]	59.3	63.0	–	
	2015	Zhang et al. [44]	78.1	65.1	–	
	2019	Liu et al. [116]	81.5	71.1	–	
	2016	Zhang et al. [50]	71.0	72.4	–	
	2020	Xue et al. [121]	82.4	78.3	–	
	2019	Liu et al. [116]	12.0	32.6	–	
	2018	Shen et al. [120]	79.2	82.2	–	–
	2018	Liu et al. [116]	82.2	73.3	–	
	2015	Zhang et al. [44]	81.9	77.9	–	
Shanghai Tech. A	2019	Liu et al. [116]	86.5	69.7	–	
	2018	Idrees et al. [43]	79.0	72.3	–	
	2016	Zhang et al. [50]	76.5	81.7	–	
	2020	Xue et al. [121]	87.3	79.2	–	
	2019	Liu et al. [116]	15.6	37.5	–	
	2019	Liu et al. [116]	79.1	60.1	–	
	2018	Shen et al. [120]	80.2	78.8	–	–
	2018	Liu et al. [116]	75.4	79.3	–	
Shanghai Tech. B	2015	Zhang et al. [44]	84.1	75.8	–	
	2019	Liu et al. [116]	78.1	73.9	–	
	2019	Idrees et al. [43]	76.8	78.0	–	
	2019	Zhang et al. [50]	82.4	76.0	–	
	2020	Xue et al. [121]	86.7	80.5	–	
	2019	Liu et al. [116]	60.0	23.0	–
	2019	Liu et al. [116]	73.7	79.6	–	
	2018	Shen et al. [120]	68.5	81.2	–	–
	2018	Liu et al. [116]	73.8	78.2	–	
World Expo	2015	Zhang et al. [44]	79.5	73.1	–	
	2019	Liu et al. [116]	71.6	75.4	–	
	2019	Idrees et al. [43]	72.4	78.3	–	
	2019	Zhang et al. [50]	80.9	77.5	–	
	2020	Xue et al. [121]	82.0	81.5	–	

**Table 3:** CMS performance in the form of mean absolute error (MAE) and mean squared error (MSE).

Year	Database	Year	MAE	MSE
	Pandey et al. [46]	2020	94	104
	Kumagai et al. [122]	2017	361	493
	Sindagi et al. [123]	2017	322	341
	Li et al. [96]	2017	266	397
	Sam et al. [124]	2017	318	439
	Han et al. [125]	2017	196	156
	Yao et al. [123]	2017	322	341
	Zhang et al. [50]	2016	377	509
	Walach et al. [95]	2016	364	341
Kumbh Mela	Marsden et al. [126]	2016	126	173
	Hu et al. [114]	2016	137	152
	Rodriguez et al. [55]	2015	655	697
	Onoro et al. [127]	2015	333	425
	Zhang et al. [44]	2015	467	498
	Liu et al. [120]	2014	197	273
	Idrees et al. [43]	2013	419	541
	Chen et al. [39]	2012	207	246
	Lempitsky et al. [56]	2010	493	487
	Ding et al. [128]	2020	1.4	2.0
	Ding et al. [48]	2018	1.4	2.0
BRT	Kumagai et al. [122]	2017	1.7	2.4
	Zhang et al. [50]	2016	2.2	3.4
	Pandey et al. [46]	2020	2.05	4.93
	Li et al. [96]	2017	1.1	1.4
	Sam et al. [124]	2017	1.6	2.1
UCSD	Zhang et al. [50]	2016	1.0	1.3
	Onoro et al. [127]	2016	1.5	3.1
	Zhang et al. [44]	2015	1.6	3.3
	Ding et al. [128]	2020	1.8	2.3
	Pandey et al. [46]	2020	4.09	14.9
Mall	Liu et al. [120]	2019	2.4	9.8
	Wang et al. [38]	2016	2.7	2.1
	Xu et al. [81]	2016	3.2	15.5
	Pham et al. [40]	2015	2.5	10
	Ding et al. [128]	2020	309	428
	Pandey et al. [46]	2020	483	397
	Li et al. [96]	2017	266	397
	Zhang et al. [50]	2016	338	424
	Zhang et al. [44]	2015	338	424
UCF_CC_50	Onoro et al. [127]	2016	465	371
	Sam et al. [124]	2017	306	422
	Onoro et al. [127]	2016	465	371
	Sam et al. [124]	2017	306	422
	Walach et al. [95]	2016	364	341
	Marsden et al. [126]	2016	338	424
	Sindagi et al. [123]	2017	310	397
	Ding et al. [128]	2020	69	114
	Pandey et al. [46]	2020	179	232
	Li et al. [96]	2017	68	115
Shanghai Tech (Part A)	Zhang et al. [50]	2016	110	173
	Zhang et al. [44]	2015	181	277
	Sam et al. [124]	2017	90	135
	Marsden et al. [126]	2016	128	183
	Sindagi et al. [123]	2017	101	152
	Ding et al. [128]	2020	10	14
	Pandey et al. [46]	2020	43	67
	Li et al. [96]	2017	20	31
	Zhang et al. [50]	2016	23	33
Shanghai Tech (Part B)	Zhang et al. [44]	2015	32	49
	Sam et al. [124]	2017	10	16
	Marsden et al. [126]	2016	26	41
	Sindagi et al. [123]	2017	20	33
	Ding et al. [128]	2020	8	–
	Pandey et al. [46]	2020	18	–
	Li et al. [96]	2017	8	–
	Zhang et al. [50]	2016	11	–
World Expo	Zhang et al. [44]	2015	12	–
	Sam et al. [124]	2017	9	–
	Shang et al. [98]	2016	11	–
	Sindagi et al. [123]	2017	8	–
	Chen et al. [39]	2012	16	–

**Table 4:** Real time CMS, year wise development.

Year	Reported Paper	Apporach Used	Task Performed
	Fiaschi et al. [15]	Regression	Density estimation
	Pandey et al. [46]	deep learning	counting
2020	Zhu et al. [41]	deep learning	counting
	Ding et al. [128]	deep learning	density estimation
	Wang et al. [47]	deep learning	counting
	Li et al. [45]	deep learning	counting
	Alotibi et al. [13]	Deep learning	counting
	Alabdulkarim et al. [3]	Deep learning	
	Bharti et al. [19]	Deep learning	counting
2019	Mohamed et al. [4]	deep learning	counting
	Tripathi et al. [61]	deep learning	counting and localization
	Liu et al. [116]	deep learning	density estimation
	Liu et al. [129]	deep learning	density estimation
	Gao et al. [115]	regression	counting
	Miao et al. [97]	deep learning	counting
	Al-Ahmadi et al. [25]	detection	counting
	Majid et al. [8]	deep learning	counting and density estimation
	Idrees et al. [49]	deep learning	density estimation
	Li et al. [96]	deep learning	density estimation
2018	Motlagh et al. [11]	regression	density estimation
	Al-Sheary et al. [12]	detection	counting
	Chackravarthy et al. [35]	deep learning and detection	counting, localization
	Sheng et al. [130]	deep learning	density estimation and counting
	Ding et al. [48]	deep learning	counting
	Lahiri et al. [62]	detection	behavier analysis
	Sam et al. [124]	deep learning	counting
	Li et al. [96]	deep learning	density estimation
2017	Martani et al. [29]	detection	localization
	Kumagai et al. [122]	deep learning	counting
	Rohit et al. [10]	detection	behavier analysis
	Fradi et al. [65]	deep learning	counting
	Rao et al. [66]	detection	counting
	Zhang et al. [50]	deep learning	counting
	Jackson et al.	Deep learning	counting
	Li et al. [26]	deep learning	counting and density estimation
	Perez et al. [29]	detection	density estimation
2016	Onoro et al. [127]	deep learning	counting
	Hu et al. [114]	deep learning	counting
	Xu et al. [81]	detection	density estimation
	Marsden et al. [126]	deep learning	density estimation
	Walach et al. [95]	deep learning	counting
	Shang et al. [98]	detection	counting and density estimation
	Zhang et al. [44]	deep learning	counting
	Giuffrida et al. [16]	deep learning	counting
	Shao et al. [51]	deep learning	density estimation
	Giuffrida et al. [16]	deep learning	counting
2015	Zhou et al. [36]	deep learning	counting and density estimation
	Danilkina et al. [37]	detection	counting
	Pham et al. [40]	deep learning	counting and density estimation
	Fu et al. [94]	deep learning	density estimation
	Ma et al. [57]	deep learning	density estimation
	Al-Salhie et al. [2]	detection	density estimation
	Jackson et al.	Deep learning	density estimation
2014	Barr et al. [32]	detection	counting
	Shao et al. [42]	detection	counting
	Idrees et al. [43]	detection	counting
2013	Chen et al. [52]	regression	density estimation
	Chen et al. [86]	detection and regression	behaviour analysis
	Jackson et al.	regression	counting
	Fiaschi et al. [15]	Regression	counting
2012	Chen et al. [39]	detection	counting and density estimation
	Song et al. [131]	regression	counting
	Garcia et al. [87]	regression	behavier analysis and density estimation
	Khouj et al. [31]	detection	counting
	Rahim et al. [5]	detection	counting and density estimation
2011	Othman et al. [6]	regression	density estimation
	Rodriguez et al. [132]	detection	counting
	Lempitsky et al. [56]	detection	counting
2010	Zainuddin et al. [23]	regression	density estimation and counting

## Abbreviations

The following abbreviations are used in this manuscript:

CV	Computer vision
CMS	Crowd Monitoring System
CCTV	Closed-circuit television
CNNs	Convolutional neural networks
DCNNs	Deep convolutional neural networks
GLCM	gray level co-occurrence matrices
HoG	Histogram of oriented gradients
IoT	Internet of Things
KLT	Kanade Lucas Tomasi
LBP	Local binary pattern
SVM	Support vector machine
MAE	Mean absolute error
MSE	Mean square error
RF	Random forest
SIFT	Scale Invariant Feature Transform
SOA	State of the art
TMLMs	Traditional machine learning methods
UAVs	Unmanned Aerial Vehicles
